# Effects of prenatal exposure to surface-coated nanosized titanium dioxide (UV-Titan). A study in mice

**DOI:** 10.1186/1743-8977-7-16

**Published:** 2010-06-14

**Authors:** Karin S Hougaard, Petra Jackson, Keld A Jensen, Jens J Sloth, Katrin Löschner, Erik H Larsen, Renie K Birkedal, Anni Vibenholt, Anne-Mette Z Boisen, Håkan Wallin, Ulla Vogel

**Affiliations:** 1 National Research Centre for the Working Environment, Copenhagen Ø., Denmark; 2 National Food Institute, Technical University of Denmark, Søborg, Denmark; 3 Institute of Public Health, University of Copenhagen. Copenhagen K, Denmark; 4 Institute for Science, Systems and Models, Roskilde University, Roskilde, Denmark

## Abstract

**Background:**

Engineered nanoparticles are smaller than 100 nm and designed to improve or achieve new physico-chemical properties. Consequently, also toxicological properties may change compared to the parent compound. We examined developmental and neurobehavioral effects following maternal exposure to a nanoparticulate UV-filter (UV-titan L181).

**Methods:**

Time-mated mice (C57BL/6BomTac) were exposed by inhalation 1h/day to 42 mg/m^3 ^aerosolized powder (1.7·10^6 ^n/cm^3^; peak-size: 97 nm) on gestation days 8-18. Endpoints included: maternal lung inflammation; gestational and litter parameters; offspring neurofunction and fertility. Physicochemical particle properties were determined to provide information on specific exposure and deposition.

**Results:**

Particles consisted of mainly elongated rutile titanium dioxide (TiO_2_) with an average crystallite size of 21 nm, modified with Al, Si and Zr, and coated with polyalcohols. In exposed adult mice, 38 mg Ti/kg was detected in the lungs on day 5 and differential cell counts of bronchoalveolar lavage fluid revealed lung inflammation 5 and 26-27 days following exposure termination, relative to control mice. As young adults, prenatally exposed offspring tended to avoid the central zone of the open field and exposed female offspring displayed enhanced prepulse inhibition. Cognitive function was unaffected (Morris water maze test).

**Conclusion:**

Inhalation exposure to nano-sized UV Titan dusts induced long term lung inflammation in time-mated adult female mice. Gestationally exposed offspring displayed moderate neurobehavioral alterations. The results are discussed in the light of the observed particle size distribution in the exposure atmosphere and the potential pathways by which nanoparticles may impart changes in fetal development.

## Background

Nanomaterial research and development is proceeding at a rapid pace and many new nanotechnology products are becoming commercially available [1]. Nanoparticles are usually defined as particles with a primary particle size between 1 and 100 nm along at least one axis. Engineered nanoparticles (ENPs) normally possess new or enhanced physico-chemical properties compared to that of the bulk material due to inherent quantum size effects, a large surface to volume ratio, and controlled particle shape and surface coating. Consequently, toxicological properties of ENPs may differ from that of their larger counterparts [2]. This highlights the need for toxicological assessment of ENPs early in material development. Free nanoparticles may behave more like a gas than solid matter because of their small size. However, most primary particles in powders are firmly agglomerated and/or aggregated.

Although primary ENPs may be emitted during production and de-agglomeration occurs during generation of dust in powder handling, subsequent re-agglomeration may still result from coagulation and scavenging when particles are aerosolized (reviewed in [3]). Consequently, it is impossible to predict the size-distribution and aerosol behavior of ENPs or their potential de-agglomeration during airway deposition. Therefore, experimental work is urgently required to assess these parameters as well as to determine the resulting biological effects *in vivo*. When inhaled, a considerable fraction of sub-μm size particles may deposit in the deeper airways. Once deposited in the lung, material may be retained for a long time [4]. Nanoparticles can also translocate across the lung epithelium, although the rate of distribution to other organs varies [3,5-7]. Airborne particles released during production or handling of ENPs are therefore of particular concern.

The toxicological properties of nanosized particles are generally poorly understood, although knowledge in some areas (especially inflammation and particle translocation) is rapidly growing. Reproductive and developmental toxicity is integrated into the nanomaterials research strategy of the U.S. Environmental Protection Agency [8] and recommended by the Reproductive Health Research Team under the National Occupational Research Agenda of the U.S. National Institute of Occupational Safety and Health [9]. Nanomaterials may affect the developing fetus either directly or indirectly. Direct effects might occur after translocation of particles from maternal lung to blood and then across the placenta. By the indirect pathway, maternal pulmonary inflammation orchestrates release of signaling molecules which potentially affect both mother and fetus. Preliminary work suggests that the fetal nervous system is specifically sensitive to maternal particulate exposure during pregnancy [10,11]. Today very little is known on developmental toxicity of nanomaterials.

Titanium dioxide (TiO_2_) has previously been used as a generic model compound to illustrate potential toxic effects of exposure to relatively inert nanoparticles. However, TiO_2 _is also a widely-used industrial nanomaterial (e.g., sunscreens and lacquers with "invisible" UV-filters, and paints with photocatalytic-induced self-cleaning properties). Thus, the exposure of consumers and factory workers who handle TiO_2 _nanomaterials and nanomaterial-based products must be considered. Increasing evidence suggests that the toxicity of TiO_2 _not only depends on size, but also varies with crystalline polymorph, particle shape, surface coating and functionalization (reviewed in [12]). Thus silica-coated TiO_2 _increased lung inflammation significantly compared to pure TiO_2 _and pure silica in the mouse [4].

The present study investigated developmental neurotoxicity in offspring of mice that inhaled TiO_2 _(UV-titan L181, a coated and chemically modified rutile) during pregnancy, in parallel with maternal inflammatory response. Effects on the nervous system were evaluated by use of a neurobehavioral test battery. Furthermore, particle physicochemical properties and exposure were characterized in detail.

## Materials and methods

### Animals

Time-mated, nulliparous mice (C57BL/6BomTac, Taconic Europe, Ejby, Denmark) arrived at gestation day (GD) 3 and were randomly grouped 5 or 6 in polypropylene cages with bedding and enrichment (removed during nursing). Animals were housed under controlled environmental conditions, with 12 hour light from 6.00 a.m. and access to food (Altromin 1324) and tap water ad libitum (further information in Additional file 1). On GD4, animals were weighed and assigned to two groups of 22 and 23 animals, respectively, with similar weight distributions. For cross-over mating, naïve CBA/J mice (Charles River Wiga, Sulzfeld, Germany) were supplied at nine weeks of age. Procedures complied with EC Directive 86/609/EEC and Danish regulations on experiments with animals (Permission 2006/561-1123).

### Material characterization

This study used UV-titan L181 (Kemira, Pori, Finland), a rutile modified with unspecified amounts of zirconium (Zr), silicon (Si), aluminum (Al) and coated with polyalcohols.

Physical particle size, morphology and general state of agglomeration/aggregation were determined by analysis of particles suspended on holey carbon-coated Cu TEM-grids using a 200 kV Transmission Electron Microscope (TEM) (Tecnai G20, FEI Company, Hillsboro, Oregon, USA). Sample preparation for TEM analysis is described in Additional file 1.

Crystalline phases and crystallite sizes were determined by powder X-ray diffraction (XRD) with a Bruker D8 Advance diffractometer equipped with a Lynxeye CCD detector (Bruker AXS Inc., Madison, WI 53711-5373, USA), using monochromated Cu_Kα1 _(1.540598 Å) rays. Results were obtained by Rietveld refinement of the X-ray diffractograms using Bruker TOPAS V4.1 software. Elongation was determined by analysis of reflections from principal crystallographical axis using the Scherrer equation.

Specific surface area was determined on a Quantachrome Autosorp-1 (Quantachrome GmbH & Co. KG, Odelzhausen, Germany) using multipoint Brunauer, Emmett, and Teller (BET) nitrogen adsorption method after 1 h degassing at 300°C. Analysis was completed according to DIN ISO 9277 as a commercial service by Quantachrome GmbH & Co. KG.

Elemental composition was analyzed by X-ray Fluorescence analysis on a Philips PW-2400 spectrometer as a commercial service by the Department of Earth Sciences, University of Aarhus, Denmark. Elemental concentrations were determined using their standard protocol using rock standards for calibration.

The organic coating of the UV-titan particles was extracted with methanol by Pressurized Liquid Extraction (PLE) at 2000 psi and 200°C, followed by centrifugation at 4000 rpm (3310 g) for 10 min. Chemical composition of the supernatant was analyzed by laser desorption ionization and time of flight MS (MALDI-TOF without matrix) on a stainless steel ground target with a Bruker AutoFlex II (Bruker Daltonics, Inc., Bremen Germany). Acurate mass determination (1 ppm) was performed with electrospray-MS (ESI-MS) on a Bruker microQ-TOF (Bruker Daltonics, Inc., Bremen Germany) with direct injection. The masses are reported as mass to charge ratios (*m/z*) of the protonated compounds ([M+H]^+^).

### Exposure

Mice were exposed to filtered clean air or a target concentration of 40 mg UV-Titan/m^3 ^on GD8-18, one hr/day as described [13,14]. Airflow in the exposure chamber was dynamic (20 L/min) with evenly distributed exposure atmosphere. A microfeeder aerosolized powder particles through a dispersion nozzle at a pressure of 5 bar (Fraunhofer Institute für Toxicologie und Aerosolforschung, Hannover, Germany). The dose from one hour exposure to 40 mg TiO_2_/m^3 ^corresponds to the 8-hr time weighted average (TWA) occupational exposure limit according to Danish Regulations [15]. Animals were placed separately in rooms of a "twelve-room-pie"; a cylindrical wire mesh cage (∅ 29 cm, height 9 cm) with radical partitions. Females were observed for signs of toxicity and returned to cages less than 5 min after exposure. Body weight was recorded before exposure on GD9, 11, 14, and 18.

### Exposure monitoring

Mass-concentrations of total suspended dust was controlled periodically by filter sampling and adjusted to maintain a concentration of ~40 mg/m^3^. Exposure air was sampled on pre-weighed Millipore Fluoropore filters (∅ 2.5 cm; pore size 0.45 μm) at an airflow of 2 L/min using Millipore cassettes, for 10 min. Filters were weighed immediately on a Sartorius Microscale (Type M3P 000V001). Final gravimetric data were obtained on acclimatized filters (50%RH and 20°C).

Particle number and size distribution in the exposure atmosphere were monitored using a GRIMM Sequential (Stepping) Mobility Particle Sizer (SMPS) system for sub-μm particles (12.8 to 486 nm; based on the rutile density of 4.25 g/cm^3 ^[16]) and a GRIMM Dustmonitor (Model 1.106) for coarse particles (0.75 to > 15 μm). The SMPS consisted of a Long Electrostatic Classifier (Model No. 5.521) and a GRIMM Condensation Particle Counter (Model 5.400). The time resolution was 218 and 6 s for the SMPS and Dustmonitor data, respectively (see Additional file 1 for further explanation on the on-line particle exposure monitoring).

### Parturition and lactation

After exposure on GD18, females were singly housed. Delivery was expected on GD20, and designated postnatal day (PND) 0. Pups were counted and sexed on PND1. Dams and individual pups were weighed at PND1, 8, 11, 16, 19, and 22. On PND2, one pup from litters with at least 5 pups, and on PND23-24 one male and one female per litter, were sacrificed by decapitation. Lungs, liver, heart, brain, and on PND2, stomachs containing milk, were dissected, weighed, snap frozen in liquid N_2 _and stored at -80°C. At weaning (PND22), one male and female per litter were randomly chosen for behavioral testing and housed as described.

Non-pregnant time-mated females without implantations ("NP females") were euthanized on PND3 (i.e. 5 days post exposure) and subjected to bronchoalveolar lavage (BAL), as were dams with litters at PND24-25 ("P females"; 26-27 days post exposure). Females were anaesthetized with Hypnorm and Dormicum and sacrificed by withdrawal of heart blood (stabilized in 0.17 mol/l K_2_EDTA). BAL was performed as described below, followed by determination of uterine implantation sites and dissection of organs as described for offspring.

### Titanium in tissue and milk

Approximately 25-75 mg tissue (lung and liver for adults, liver for offspring) and 110-140 mg (milk) were weighed and analyzed for content of titanium (Ti). For PND2 pups, milk and liver samples were pooled from 4-5 animals. Maternal lung was included to determine remaining TiO_2 _and liver to assess systemic distribution in adults [17,18] and fetal animals [19]. Tissues were digested in concentrated nitric acid (PlasmaPure, SCP Science, Quebec, Canada) in a microwave oven (Multiwave, Anton Paar, Graz, Austria), and Ti content determined by quadrupole-based inductively coupled plasma mass spectrometer (ICPMS 7500ce, Agilent Technologies, Tokyo, Japan) equipped with a collision/reaction cell (CRC). The CRC was pressurized with helium as collision gas to reduce polyatomic interferences on Ti isotopes. Settings for ICPMS measurements are given in (Additional file 1, Table S1). Sulphur-containing polyatomics (e.g. ^32^S^16^O^+^) strongly interfered with the most abundant Ti isotope, ^48^Ti (abundance 73.8%). There was less interference with ^49^Ti and ^50^Ti (abundance 5.5 and 5.4%, respectively), which were selected for quantitative analysis. The limit of detection (LOD) for Ti in tissues, based on three times the standard deviation of repeated blank measurements, was estimated to be 0.2-5 mg/kg depending on sample intake and dilution.

### BAL preparation and analyses

We used BAL cell composition and neutrophil influx to indicate lung inflammation. This has proven to be a relevant and sensitive marker of pulmonary inflammation (e.g. [20,21]). BAL was performed four times with 0.8 ml 0.9% sterile saline ([20]; further information in Additional file 1). The total number of cells and of dead cells in BAL samples was determined in cell suspension B by NucleoCounter. Differential counts of macrophages, neutrophils, lymphocytes, eosinophils, and epithelial cells were determined by counting 200 cells in cell supernatant fixed with 96% ethanol and stained with May-Grünwald-Giemsa stain. All slides from both time points were randomized, blinded and scored on the same day. Total number of cells was calculated by combining data from differential cell counts with the total number of cells in BAL.

### Behavioral testing

Investigations were performed during the light period. Exposed and control animals were tested alternately. Animals were transferred to the experimental room 1 hr before the first test. Observers were blinded to exposure status of the animals, and the same observer was used throughout any specific test.

Learning and memory was tested in the Morris water maze at age 11 and 15 weeks (males), and 12 and 16 weeks (females) as described [13] with minor modifications. A stable, invisible platform was submerged 1 cm below the water surface in a circular plastic pool (∅ 100 cm). Animals were tested in four daily trials. Mice were placed at the designated starting position and completed the trial when climbing onto the platform. When failing to locate the platform within 60 s, animals were led to the platform. All animals spent 15 s on the platform before returning to the cage. The following scheme was used: *Learning: *Test for 5 consecutive days with platform in center of the southeastern quadrant. *Memory: *Three weeks later, test with platform in south-eastern quadrant, for 3 days. *Reversal learning: *The following day, test with platform in north-western quadrant for 4 trials. *New learning: *The following day, test with platform in center of pool for 4 trials. Noldus Ethovision (Version 5, Noldus Information Technology, Wageningen, The Netherlands) was used to register latency and path length, and calculated swimming velocity and relative occupancy in the each of the quadrants.

Activity was assessed for 3 min at 14 weeks of age in an open field using the dry water maze pool. Trials commenced in the center of the field and the location of the animal was registered by Noldus Ethovision XT version 5. The tracking device calculated total ambulation, which was subsequently split into three time-bins of 1 min to test for habituation. Duration in the central and the outer 9 cm peripheral zone of the field, as well as the number of crossings from the outer to the central zone were extracted.

Acoustic startle reaction (ASR) and prepulse inhibition (PPI) were tested at 4 months as described [22] in two chambers (San Diego Instruments, San Diego, USA) with 70 dB(A) white background noise. A piezoelectric accelerometer transduced displacement of test tubes (∅ 3.6 cm) in response to movements of the animal. Animals were acclimatized for 5 min in the tube before sessions started and ended with 5 startle trials of 40 ms 120 dB(A) bursts of white noise. In between, 35 trials were delivered in semi-randomized order (10 trials of 120 dB(A); 5 each of 4 prepulse + startle trials (prepulses of 72, 74, 78, and 86 dB(A)); 5 trials with only background noise). Tube movements were averaged over 100 ms following onset of the startle stimulus (AVG). The five AVGs for each prepulse intensity were averaged and used to calculate PPI, which was expressed as percent reduction in AVG compared to the average of the 10 middle startle trials: %PPI = 100*((AVG at prepulse+startle trial)/(AVG at startle trial))*100%.

### Time-to-first F2 litter

At 19 weeks of age, control and exposed offspring were cross-mated to naïve CBA/J mice (12 weeks old) and time-to-first-delivery of F2 litter, litter size, and gender ratio were recorded.

### Statistics

Litter was considered the statistical unit. Gestational parameters were analyzed by Mann-Whitney *U*-test, and time-to-first-delivery by log rank test (separately by gender). ANOVAs were applied to the remaining data when relevant with repeated measures in trials, days, or time-bins. In the analysis of weight gain in adult females, Ti in adult tissues, and BAL cell counts, the factor "Pregnancy" was added to distinguish (barren) NP females from (littering) P females. Since these groups of adult females differed with regard to both time after exposure and pregnancy, only pairwise comparisons related to exposure were explored. ANCOVA controlled for litter size in the analyses of weight gain during exposure, birth weights, and pre-weaning pup weights. Behavioral data were analyzed by two-way ANOVA, with Prenatal exposure and Gender as factors, apart from startle data, where PPI was analyzed separately for each prepulse intensity [22]. Pairwise comparisons were performed by T-test or Mann Whitney U-test (*p *< 0.1). Analyses were performed in SYSTAT Software Package 9, MINITAB 14, and SAS 9.1.

## Results

### Particle characteristics

Physicochemical characteristics of the UV-titan sample are summarized in Table 1. Rutile was the only crystalline phase in the sample and TiO_2 _accounted for 70.8 wt%. Residual mass was composed of Zr, Si, Al, and a little sodium (Na) as well as 5.2 wt% volatiles (loss on ignition). Stochiometric calculations show that the modifier elements partly occurred in oxides, but presence of native metals or non-stochiometric amorphous compounds are also possible. BET measurements show that the specific surface area was ~38 m^2^/g higher (i.e. 107.7 m^2^/g) than reported by the manufacturer (ca. 70 m^2^/g). This difference in specific surface area may arise due to out-gassing of the powder at 300°C for 1 h before analysis. This may have volatilized the organic coating, thereby increasing the accessible surface area.

**Table 1:** Physico-chemical characteristics of UV-Titan L181 particles.

	This study	Product data sheet
Phases	Rutile	Rutile
Average XRD-size [nm]	20.6 ± 0.3	Approx. 17
XRD-size [100]^a^	14.4-15.5	-
XRD-size [001]^a^	38.4	-
Specific surface area [m^2^/g]	107.7	Approx. 70
Elemental concentrations	[wt%]	
Silicon	5.61	-
Titanium	42.44	-
Aluminum	2.42	-
Zirconium	8.65	-
Sodium	0.45	-
Oxygen^b^	35.24	-
LOI	5.19	-
TGA	6.1 ± 0.4	-

LOI, loss on ignition; TGA, thermogravimetric analysis (N_2 _atmosphere, 40 - 800°C, 10°C/min). ^a ^Estimate of the average crystallite size along the shortest and longest crystallographic direction. ^b^Calculated by difference from 100 wt%.

**Figure 1:**
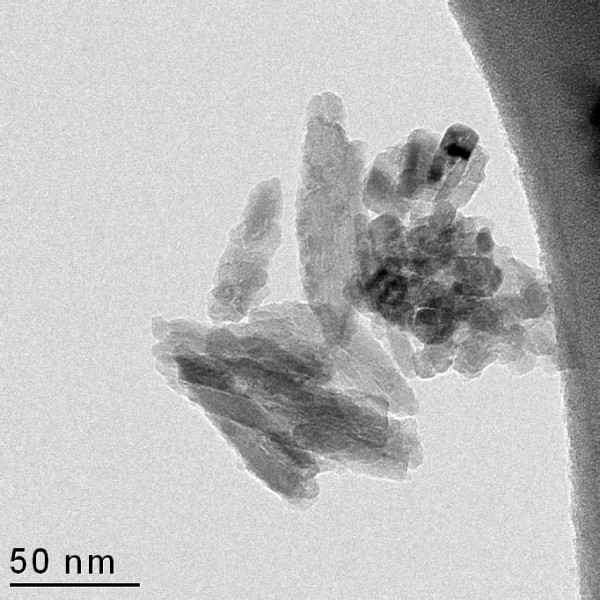
**TEM image of TiO_2 _crystallites**. Transmission electron microscopy image showing the typical equidimensional to elongated morphology of the TiO_2 _crystallites in UV-Titan L181. Bar = 50 nm.

By TEM, we mainly observed aggregates and agglomerates of equidimensional to needle-shaped TiO_2 _crystallites with diameters ranging from less than 10 nm to more than 100 nm along the shortest and longest axis, respectively (Figure 1). The average crystallite size was determined to be 20.6 ± 0.3 nm, in reasonable agreement with product data (Table 1). However, calculation of the average crystallite sizes in specific crystallographic directions indicated that the size along the c-axis (38.4 nm) was about 2.5 times the average size along the × and y (short) axes (14.4-15.5 nm). This is supported by the TEM analysis (Figure 1).

The organic coating was analyzed by MALDI-MS. The identity of positive (protonated) molecules was deduced from the *m/z *values. The observed molecular formulas of the tentatively identified compounds are summarized in Table 2. The *m/z *values occurred in 4 series (as indicated in the MALDI-TOF spectrum in Additional file 1, Figure S1). Within each series, compounds were spaced by an *m/z *of 16, corresponding to oxygen, as shown in Table 2. This indicates that within each series, similar structures only differ by an OH-group. Thus, compounds with m/z = 104, 113, 115 and 173 contain at least two OH-groups. Furthermore, compounds in group 1 contain one c-c double bond, in group 2 three double bonds, in group 3 two double bonds and in group 4 three double bonds or carbonyl groups. In ESI-MS, only one peak was observed at *m/z *= 157 and this is the only peak for which the molecular formula has been determined via accurate mass determination. Our mass spectrometric analyses suggest that a fraction of the UV Titan L181 consists of polyalcohols with a chain length of 4, 6 or 8 carbons. However, these polyalcohols appear to be of a complex nature.

**Table 2:** Observed m/z values and tentative molecular formulas

	m/z **[M+H]**^+^	Tentative molecular formular
1	72	C_4_H_7_N
	88	C_4_H_7_NO
	104	C4H7NO2
2	81	C_6_H_8_
	97	C_6_H_8_O
	113	C_6_H_8_O_2_
3	83	C_6_H_10_
	99	C_6_H_10_O
	115	C_6_H_10_O_2_
4	141	C_8_H_13_O_2_
	157*	C_8_H_13_O_3_
	173	C_8_H_13_O_4_

* Molecular formula determined from exact mass measurement (mass accuracy 1 ppm)

### Exposure characteristics

**Figure 2:**
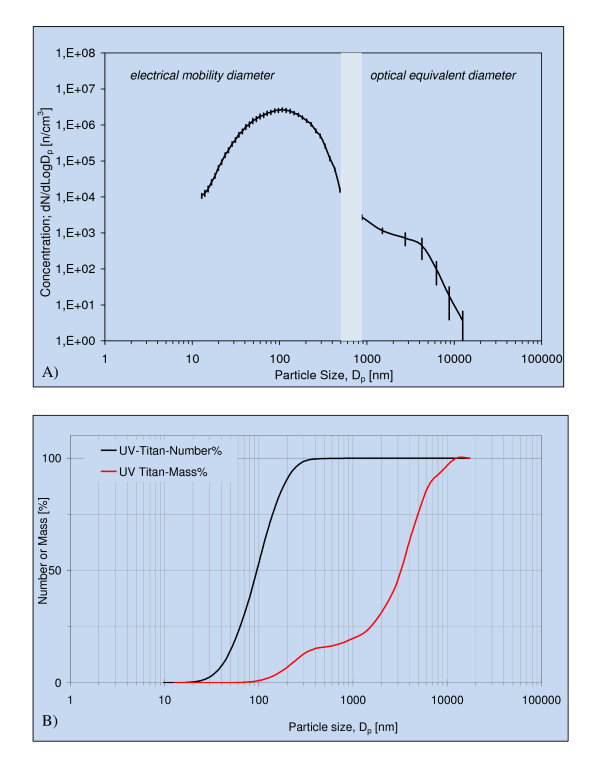
**Characteristics of the exposure atmosphere**. A) Particle number size distribution of the UV-Titan L181 in the exposure chamber. Data are based on nine one-hour exposure measurements. Mean ± SD. B) Accumulated number and mass concentration of particle concentrations in the exposure chamber. It is assumed that the optical and mobility particle sizes can be directly compared and data gap is filled by linear interpolation.

Filter measurements demonstrated that animals were exposed to a mean total suspended particle mass concentration of 42.4 ± 2.9 (SEM) mg/m^3 ^UV-Titan. The particle number concentration in the exposure atmosphere was 1.70 ± 0.20·10^6^/cm^3^. The major particle size-mode was ~100 nm (geometric mean number diameter 97 nm), with a coarser size mode at ~4 μm (Figure 2A). Smaller size modes were observed at ~20 nm and 1 μm. By number, 80% of the particles were between 40 and 200 nm and no particles were coarser than 12.5 μm detected (Figure 2B). The mass-size distribution was strongly dominated by μm-size particles (geometric mean 3.2 μm) and 75% of the mass were represented by particles larger than 1.6 μm (Figure 2B). The fraction of sub-100-nm-size particles amounted to 1% of the mass.

### Ti concentration in tissues and milk

Ti concentration in tissue and milk samples is shown in Table 3. Lungs from exposed females contained 38 mg Ti/kg on day 5 after the exposure and 33 mg Ti/kg on days 26-27. No Ti was detected in unexposed female lungs (*p *= 0.0002). Values were similar between control and exposed animals for all other samples.

**Table 3:** Titanium concentration in livers, lungs and milk.

Origin	Tissue	Treatment	N	Time after exposure (days)	Ti (mg/kg)
Adult females	Lungs	Exposed	3	5	38 ± 6
		Controls	3	5	< 5
		Exposed	3	26-27	33 ± 18
		Controls	3	26-27	< 0.7
	Livers	Exposed	3	5	< 0.5
		Controls	3	5	< 0.5
		Exposed	3	26-27	0.5 ± 0.3
		Controls	3	26-27	< 0.2
Pups	Livers	Exposed	2^a^	5	< 0.4
		Controls	2^a^	5	0.4 ± 0.1
		Exposed	3	26-27	< 0.4
		Controls	3	26-27	< 0.4
	Milk	Exposed	2^b^	5	< 1
		Controls	2^b^	5	< 1

Mean ± SD corresponding to the two detected Ti isotopes. Pooled sample from 5^a ^and 4^b ^animals.

### Maternal and litter parameters

Similar numbers of control and exposed females delivered litters, and none of the time-mated females without litters displayed implantations. Gestational and litter parameters were similar, apart from a slight decrease in pup viability in TiO_2 _litters (*p *= 0.083, c.f. Table B, Additional file 1, Table S2l). Only maternal lung weight showed overall statistical significant variation with exposure, in both absolute (*p *= 0.04) and relative (*p *= 0.05) measures (data not shown). Pairwise comparisons showed both measures to be marginally increased in only in P females (0.05 <*p *< 0.1). No effects related to exposure were detected for offspring organ weights.

### Lung inflammation in time-mated females

**Figure 3:**
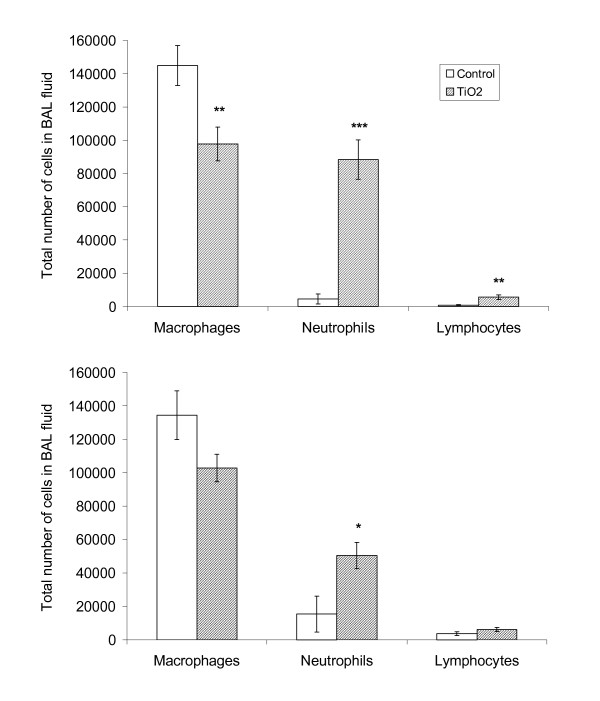
**Differential cell count in bronchoalveolar lavage fluid**. The total number of cells in BAL subdivided by cell type. A: time-mated mice that had not achieved pregnancy, 5 days after termination of exposure (n = 8-9). B: littering time-mated dams after weaning, 26-27 days after exposure (n = 10-14). Mean ±SEM. *p < 0.05, **p < 0.01, and ***p < 0.001 vs. controls.

Lung inflammation was evaluated by cell counts of BAL fluid (Table 4 and Figure 3). Overall, more neutrophils were present in BAL in TiO_2 _exposed compared to unexposed females (*p *< 0.001), with significant exposure-pregnancy interaction (*p *= 0.02). BAL from exposed NP females contained 19 times more neutrophils in BAL than did unexposed NP females (5 days after exposure, *p *< 0.001). The exposed P females displayed 3-fold more neutrophils compared to unexposed P females (26-27 days after exposure, *p *= 0.02). The exposure also resulted in overall change in macrophages (p = 0.002) and lymphocytes (p = 0.007) compared to unexposed P females. In NP females, pairwise comparisons revealed fewer macrophages (p = 0.009) but more lymphocytes (p = 0.008) in exposed compared to UNexposed NP females.  No cell type showed significant change in exposed P females compared to respective controls. Overall, a statistically significant increase in the total number of dead cells in BAL fluid (*p *= 0.03) was observed in BAL from the exposed P females (*p *= 0.004) but not in BAL from exposed NP females. Total cell counts, total number of eosinophils, and epithelial cells in BAL were did not vary with exposure.

**Table 4:** Total cell counts after bronchioalveolar lavage

Treatment	Days after exposure	Total live cell count	Dead cell count
Control	5	166500 ± 13642	14000 ± 2236 (9%)
Exposed	5	202000 ± 18083	18667 ± 2014 (10%)
Control	26-27	171600 ± 19724	13600 ± 3748 (9%)**
Exposed	26-27	177000 ± 14325	25857 ± 3141 (15%)

Cells were counted in bronchoalveolar lavage fluid from time-mated mice that had not achieved pregnancy 5 days after termination of exposure (NP; n = 8-9) and in littering time-mated dams after weaning, 26-27 days after exposure (P; n = 13-14). Mean ± SEM. **p < 0.01 vs. control P.

### Behavioral data

In the Morris water maze, no change was observed in performance as a result of prenatal TiO_2 _exposure in either male or female offspring (data not shown).

**Figure 4:**
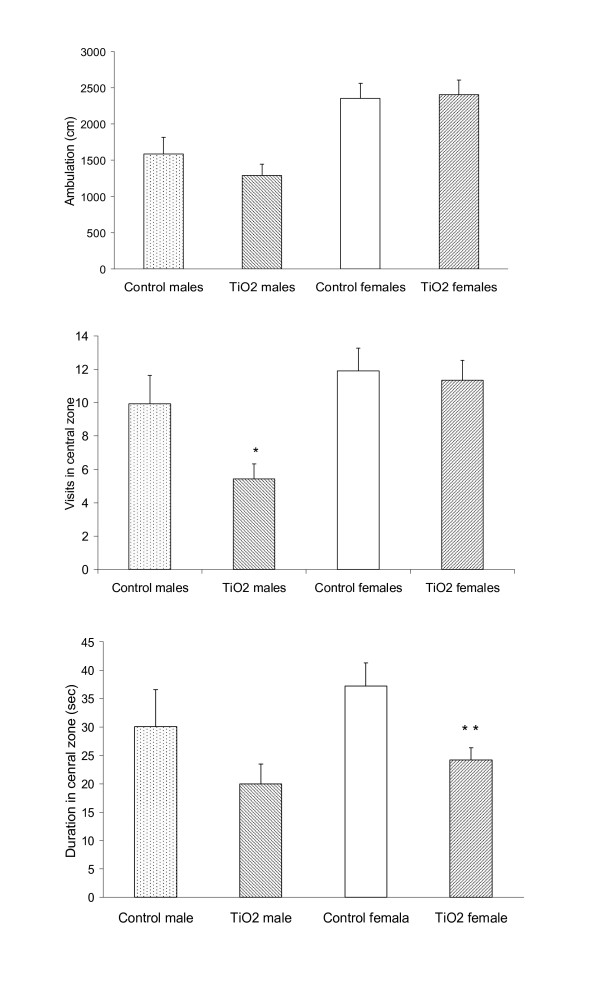
**Open field**. Open field performance during a 3-min observation period in male and female offspring from dams exposed to ambient air or TiO*_2 _*during gestation. (A) Ambulation. (B) Visits to the central zone of the open field. Time spent in central zone of the open field (C). Mean ± SEM, n = 12-14. *p < 0.05, **p < 0.01, vs. same gender controls.

In the open field, ambulation differed by gender (*p *< 0.001) but not exposure, as females moved approximately 50% longer than males (Figure 4A). Prenatally exposed animals spent significantly less time than controls in the central zone of the field (*p *= 0.009), and visited the central zone less frequently (Exposure: *p *= 0.056; Gender: *p *= 0.003). Exposed males entered the central zone significantly less frequently than unexposed males (Figure 4B, *p *= 0.021) and exposed females spent less time in the central zone than did unexposed females (Figure 4C, *p *= 0.009).

**Figure 5:**
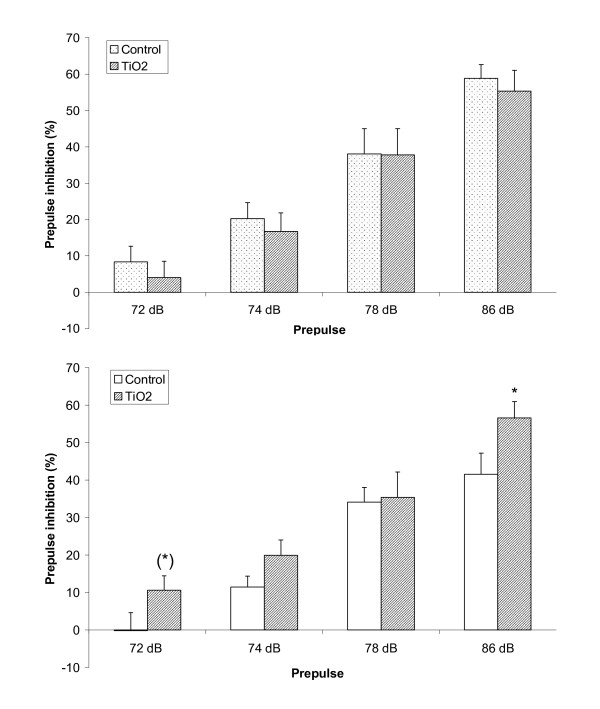
**Prepulse inhibition**. Prepulse inhibition in male (A) and female (B) offspring from dams exposed to ambient air or TiO*_2 _*during gestation, at four different levels of prepulse. Mean ± SEM, n = 10-14. (*) 0.05 <*p *< 0.1; *p < 0.05 vs. controls at same level of prepulse.

Analysis of acoustic startle demonstrated that exposed male offspring startled less than control males and were less inhibited by prepulse, whereas the opposite pattern was apparent for female offspring (Additional file 1, Figure S3). Statistical analysis substantiated a stronger PPI in prenatally exposed females at the highest and lowest prepulse compared to control offspring (Figure 5B; *p *= 0.041 and *p *= 0.089, respectively).

### Time-to-first F2 litter

**Figure 6:**
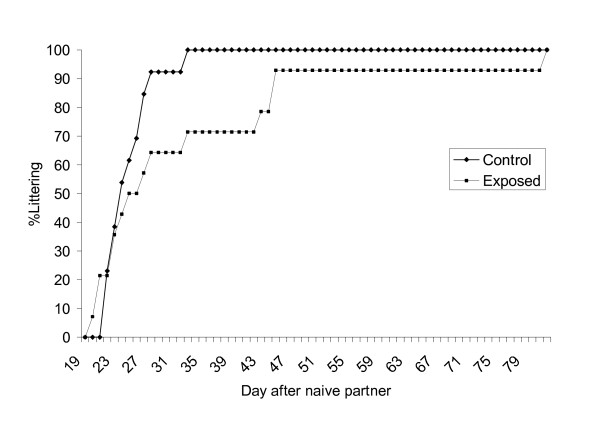
**Time-to-first F2 litter**. Littering curves for male offspring of control and UV-titan exposed pregnant mice. As adults, male C57BL offspring were mated to naïve CBA/J mice and time-to-first-delivery of F2 litter was recorded.

At termination of behavioral testing, control and exposed C57BL offspring were cross-mated to naïve CBA/J mice. Time-to-first-delivery of F2 litter was similar in control and exposed female offspring, but was extended in exposed male compared to control male offspring (32.9 ± 3.1 (SD) and 25.2 ± 16.8 (SD) days, respectively; Figure 6). However, this result did not reach statistical significance (*p *= 0.12). Litter size was similar in control and exposed F2 litters.

## Discussion

The effects of maternal inhalation of the UV Titan on offspring development were investigated. Eleven days of inhalation was associated with Ti deposition in pulmonary tissues and lung inflammation in adult females. High amounts of Ti and lung inflammation persisted in lungs 26-27 days following the last exposure. In addition, male and female mice exposed during fetal life displayed neurobehavioral alterations in adulthood. These observations occurred after a relevant route of exposure (inhalation) and dose (the 8-hour TWA for Danish Regulations). Thus, the results warrant careful scrutiny.

Our findings support previous evidence demonstrating long-term pulmonary inflammation following inhalation of TiO_2 _nanoparticles in both mice and rats [18,23-25]. Inflammation characterized by increased recruitment of neutrophils after inhalation of mixed anatase and rutile TiO_2 _nanoparticles (100 mg/m^3 ^for 6 hr/day for 5 days) was evident after two weeks in male rats, with slight signs of recovery [18,25]. A similar exposure carried out over 13 weeks also resulted in neutrophilic infiltration at 10 mg/m^3^, but not at 0.5 and 2.0 mg/m^3^. Altered cytological profiles persisted for 26 weeks in female rats and mice [24]. Interestingly it has been reported that the inflammatory response differs between the pregnant and the non-pregnant state. For example, pregnant mice displayed enhanced inflammation based on cell counts and inflammatory cytokines in BAL fluids as compared to non-pregnant mice [26]. However, the present study design did not allow determination of the relative contribution of pregnancy and time after exposure.

Inhalation of nanoparticles during pregnancy may affect fetal development, through direct or indirect mechanisms. Once in the airways, the majority of nanosized particles are predicted to deposit in the lung [7,18]. However, in the present study, despite the high number of 100 nm-size particles, the mass of airborne particles was strongly dominated by μm-size particles. Using the deposition model described in [21] and assuming electrical and optical equivalent sizes compare to the aerodynamic diameters, only 8.6% and 5.8% of the inhaled mass of air-borne UV-Titan were predicted to deposit in pulmonary and tracheobronchial regions, respectively (Additional file 1, Figure S2A). Most of the UV-Titan mass is predicted to deposit in the upper airways (42.5%) and the gastrointestinal tract (42.5%). Correspondingly, the model suggests that 56.1% of the particle number would deposit in pulmonary and 18.4% in the tracheobronchial regions (Additional file 1, Figure S2B). Only 4.1% of the particle numbers are estimated to end up in the upper airways and 4.5% in the gastro-intestinal tract.

Assuming each animal inhaled 1.8 L/hr with a particle concentration of 42.4 mg/m^3 ^through 11 exposure sessions, each animal inhaled a total of 840 μg. Applying the deposition estimated above and ignoring clearance and potential translocation, we expected a deposition of 72.5 μg in the pulmonary and 48 μg in the tracheobronchial region. The majority of the mass was expected to deposit in the gastrointestinal tract (356 μg) and skull (267 μg). Hence, with an average lung weight of 274 mg, the estimated deposited pulmonary dose amounts to 112-159 mg UV Titan/kg lung depending on whether pulmonary or bronchopulmonary regions are considered. This corresponds to 48-67 mg Ti/kg after adjusting for Ti concentration in the sample (Table 1). The lungs of females contained 38 and 33 mg Ti/kg at 5 and 26-27 days post-exposure, respectively. Thus, approximately 60-80% of predicted pulmonary UV-Titan deposition could be accounted for. Clearance from the airways is the most plausible explanation for the observed discrepancy. In a recent study, 60% of the deposited 10 × 40 nm-size Si-coated rutile were cleared from lungs of mice inhaling 10 mg/m^3 ^for a total of 32 h over a 4 week period [4], which is in line with the present findings.

A small fraction of inhaled particles may translocate from the lungs to maternal body compartments [5,18]. Results from systemic exposure by intravenous injection suggest that most nano-size TiO_2 _is distributed to the liver in rodents [17,18,27]. However, less than 0.25% of inhaled 20-30 nm mixed anatase/rutile (20 hr inhalation of 100 mg/m^3^) was detected in liver up to 19 days after exposure, although some deposition in mediastinal lymph nodes was noted [18]. The low hepatic Ti-concentrations in the present study also suggest negligible translocation to the liver. Considering the observed particle size of the UV-Titan sample (Figure 1 and 2), translocation may only be relevant for a small fraction of the particles. About 30% of the particle number, but only 0.75% of the weight of the inhaled UV-Titan particles smaller than 100 nm, were estimated to deposit in the pulmonary region. If translocation and accumulation in the liver occur at an efficiency of 0.25‰, then the Ti concentration in the liver would be very low (< 375 ng) even though the number could be on the order of 100 particles. As expected, the Ti content in the offspring liver tissue was below the limit of detection even a few days after birth.

It has been demonstrated that a very limited fraction of particles in maternal blood is expected to transfer to the fetal compartment, although translocation may be higher for smaller compared to larger nanoparticles [19,28,29]. However, recent data from human placental perfusion models showed that nearly 30% of polystyrene beads in the maternal circuit were transferred to the fetal compartment [30]. Takeda et al. (2009) also observed TiO_2 _aggregates of particles in offspring testicle and brain tissue as long as six weeks after birth when pregnant mice were exposed subcutaneously to 25-70 nm particles at a total dose of 16 mg/kg [10]. Thus, since nano-sized particles may reach fetal tissues, direct exposure of the fetus to particles is possible.

Direct exposure of the fetus in the present study is expected to be low. However, indirect mechanisms could lead to fetal effects. Indeed, developmental effects have been observed even following limited maternal exposure. For example, the offspring of mothers exposed intranasally to a single dose of 50 μg nano-sized particles (TiO_2_, carbon black, or diesel exhaust particles) during gestation display a more pronounced asthmatic phenotype. The underlying mechanism for this outcome remains unknown [26].

Engineered nanoparticles are often coated and/or organically functionalized. In this study, rutile is modified by Zr, Si, Al, and Na and coated with complex polyalcohols. Degradation or release of such coatings followed by placental transfer presents an additional mechanism by which nanoparticles may influence fetal development. Also, metals leached or dissolved from the nanomaterial may speciate into mobile ions and traverse the placenta [31,32]. For future studies it would be interesting to investigate the effect of pure and coated particles to elucidate the role of the particle surface in toxicity.

Thus, the literature, as well as our results, suggests that signaling cascades may be responsible for effects in animals exposed *in utero*. This is corroborated by observations of widespread changes in the expression of genes associated with acute phase, inflammation and immune response in NP females in the present study (Halappanavar S, personal communication). In the present experiment, maternal lung-inflammation following inhalation of UV-Titan may have resulted in cross-placental transfer of inflammatory cytokines [33]. Also diesel exhaust has been shown to increase placental mRNA levels of inflammatory cytokines in pregnant mice [34]. It is well established that maternal inflammation may adversely interfere with fetal neurodevelopment. Thus activation of the maternal immune system (in absence of pathogens) during gestation may induce significant changes in the nervous system and behavior of the offspring, and administration of exogenous pro-inflammatory cytokines may induce structural and functional abnormalities in the adult offspring (reviewed in [33,35]). Particle-induced inflammation may therefore represent yet another pathway for interference with fetal development. Finally, postnatal transfer could potentially take place through maternal milk [32], although we detected no Ti in milk a few days after delivery.

Offspring were evaluated in a neurobehavioral test battery. Exposed offspring tended to avoid the central zone of the open field. Furthermore, exposed female offspring displayed enhanced prepulse inhibition. To our knowledge this is the first study of prenatal (inhalation) exposure to nano-TiO_2 _to assess nervous system function after birth. As described above, one study of prenatal exposure to pure 20-70 nm anatase TiO_2 _reported particle aggregates in offspring brain tissue six weeks after birth. In addition, nervous tissue (olfactory bulb) showed some indications of increased apoptosis [10]. Another study, with an almost similar prenatal exposure regimen, reported gene expression changes related to apoptosis, development, and central neural system function in whole brain homogenate [36]. Two older studies assessed function of the central nervous system after prenatal exposure, but to trace amounts of dissolved Ti rather than particles. Exposed male offspring displayed some signs of delayed reflex emergency and decreased ambulation in the open field test, whereas female offspring showed increased number of errors in a maze learning test [37,38]. However, limited information of study designs for all three studies renders interpretation of these findings difficult. The minimal database on neurodevelopment following prenatal exposure to nanoparticles does not provide a background on which gender specificity of effects can be discussed. However, it is a common observation in neurodevelopmental studies that male and female offspring display differential phenotypes after prenatal insults (e.g. [39,40]), as is also reflected in the present study.

In a previous study, prenatal exposure to 20-70 nm anatase TiO_2 _particles were observed in Leydig and Sertoli cells and in spermatids, 4 days and 6 weeks after birth. Furthermore, daily sperm production was significantly lower in exposed compared to control offspring [10]. Also exposure of pregnant mice to 14 nm carbon black particles by intratracheal instillation has been associated with significantly decreased daily sperm production and seminiferous tubule damage in the male offspring [41]. Following the behavioral testing, fecundity was therefore assessed by mating offspring to unexposed mice and recording time-to-first-litter. Male offspring that had been exposed to particulate TiO_2 _during fetal life displayed a (non-significant) delay in time-to-first-litter. With this endpoint we would recommend to increase statistical power by increasing the number of breeding pairs.

## Conclusions

Inhalation of nano-sized coated TiO_2 _induced long-term lung inflammation in time-mated adult mice, and their gestationally exposed offspring displayed neurobehavioral alterations. Exposure was conducted at an exposure level approximating the 8-hour TWA in Denmark. Future assessments of TiO_2 _toxicity would benefit from adding more dose levels to aid risk assessment. Careful analysis of physicochemical characteristics of the nanomaterial and monitoring of the exposure atmosphere made estimation of actual dose possible. Although direct fetal exposure to UV-Titan was probably low, both direct and indirect pathways resulting from the exposure may interfere with fetal development and it is likely that several pathways operate to determine the outcome. In future studies, mapping changes in e.g. the molecular pathways that are altered in the brains of the descendents would help to shed light on the biological basis for the altered behavior. This would also reveal the molecular targets of the exposure and open up for understanding the potential relevance to human health.

## Abbreviations and definitions

∅: Diameter; ANCOVA: analysis of covariance; ANOVA: analysis of variance; ASR: acoustic startle reaction; AVG: average of tube movements for 100 ms following onset of startle stimulus; BAL: bronchoalveolar lavage; BET: Brunauer, Emmett, and Teller; CRC: collision/reaction cell; dB(A): decibel, A-weighted; EC: European Commission; ESI-MS: electrospray-MS; ENP: engineered nanoparticles; F2 litter: second generation litter; GD: gestation day; ICPMS: inductively coupled plasma mass spectrometer; LDI-TOF: laser desorption ionization time of flight mass spectrometry (MALDI-TOF without matrix assistance); LOD: limit of detection; *m/z *: mass to charge ratios MBq, megabecquerel; MS: mass spectrometry; nm: nanometer; NP females: non-pregnant time-mated females without implantations; P females: time-mated females with litters; PND: postnatal day; PPI: prepulse inhibition; SD: standard deviation; SEM: standard error of the mean; SMPS: sequential (stepping) mobility particle sizer; TEM: transmission electron microscopy; TGA: thermogravimetric analysis; TiO_2_: titanium dioxide; TWA: time weighted average; UV: ultraviolet; XRD: X-ray diffraction.

## Competing interests

The authors declare that they have no competing interests.

## Authors' contributions

KSH was substantially involved in design of the study, acquisition and analysis of gestational and behavioral data, statistical analyses, interpretation of results, and drafted the manuscript. PJA was substantially involved in designing the study, acquisition of gestational data, and drafting of the manuscript regarding BAL data and revised the manuscript critically. KAJ made substantial contribution to particle analysis, drafting of the manuscript regarding exposure characterization and discussion of data, and revised the manuscript critically. JJS, EHL and KAL analyzed Ti in tissues, and drafted the manuscript regarding this endpoint. RKB carried out particulate X-ray diffraction and drafted the manuscript regarding this endpoint. AV characterized the organic coating of the UV-titan and drafted the manuscript regarding this endpoint. HW contributed substantially to the study design, set up the particulate exposure and the exposure protocol, and revised the manuscript critically. AMB carried out postnatal breeding and contributed to the manuscript regarding this endpoint. UBV contributed substantially to the study design, drafting and interpretation of BAL data, and revised the manuscript critically. All authors have read and approved the final manuscript.

## Supplementary Material

Additional file 1 **PDF-file, containing additional description of methods, two tables and three figures**. - Housing of animals. - BAL preparation and analysis. -Sample preparation for TEM analysis. - On-line particle exposure monitoring. Table S1: Settings for the ICPMS measurements. Table S2: Pregnancy and litter data. Figure S1: MALDI-TOF spectrum of methanol extract of UV-titan 181. Figure S2: Estimated deposition curves in the airways of UV-Titan in exposed mice. (A) Estimated accumulated mass deposition curves in the airways of UV-Titan in exposed mice. (B) Estimated accumulated particle number deposition curves in the airways of UV-Titan in exposed mice. Figure S3. Basal startle reaction, in male (A) and female (B) offspring from dams exposed to ambient air or TiO_2 _during gestation. Click here for file
