# Correction: Effects of prenatal exposure to surface-coated nanosized titanium dioxide (UV-Titan). A study in mice

**DOI:** 10.1186/1743-8977-8-14

**Published:** 2011-05-05

**Authors:** Karin S Hougaard, Petra Jackson, Keld A Jensen, Jens J Sloth, Katrin Löschner, Erik H Larsen, Renie K Birkedal, Anni Vibenholt, Anne-Mette Z Boisen, Håkan Wallin, Ulla Vogel

**Affiliations:** 1 National Research Centre for the Working Environment, Copenhagen Ø., Denmark; 2 National Food Institute, Technical University of Denmark, Søborg, Denmark; 3 Institute of Public Health, University of Copenhagen. Copenhagen K, Denmark; 4 Institute for Science, Systems and Models, Roskilde University, Roskilde, Denmark

## Correction

Some statements in our paper [1] are incorrect, below find corrections.

Table one, Zirkonium

The content of Zirkonium in the UV Titan was miscalculated by a factor 10. The correct number is therefore 0.86% (and not 8.65%).

P. 12, 1^st ^column (In discussion)

For calculation of the retained amount of titanium, some inconsistencies appear in the text regarding titanium vs. the studied particle UV Titan, leading to overestimation of the retained dose. The correct text and numbers are given below:

Assuming each animal inhaled 1.8 L/hr with a particle concentration of 42.4 mg/m^3 ^through 11 exposure sessions, each animal inhaled a total of 840 μg. Applying the deposition estimated above and ignoring clearance and potential translocation, we expected a deposition of 72.5 μg in the pulmonary and 48 μg in the tracheobronchial region. The majority of the mass was expected to deposit in the gastrointestinal tract (356 μg) and skull (267 μg). Hence, with an average lung weight of 274 mg, the estimated deposited pulmonary dose amounts to 263-440 mg UV Titan/kg lung depending on whether pulmonary or bronchopulmonary regions are considered. Adjusting for Ti in the sample, this corresponds to 112-159 mg Ti/kg lung (Table one). The lungs of females contained 38 and 33 mg Ti/kg at 5 and 26-27 days post-exposure, respectively. Thus, approximately 21-24% of predicted pulmonary UV-Titan deposition could be accounted for."

Page 5, 2^nd ^column (last lines of "Behavioral testing")

Final paragraph should read "The average of the 10 middle startle trials %PPI = 100-((AVG at prepulse+startle trial)/(AVG at startle trial))*100%".
