# Supplementary material for: Effects of prenatal exposure to surface-coated nanosized titanium dioxide (UV-Titan). A study in mice
Source: Part Fibre Toxicol. 2010 Jun 14;7:16. doi: 10.1186/1743-8977-7-16 (PMC2908059; doi:10.1186/1743-8977-7-16)
Supplement: Additional file 1 — PDF-file, containing additional description of methods, two tables and three figures. - Housing of animals. - BAL preparation and analysis. -Sample preparation for TEM analysis. - On-line particle exposure monitoring. Table S1: Settings for the ICPMS measurements. Table S2: Pregnancy and litter data. Figure S1: MALDI-TOF spectrum of methanol extract of UV-titan 181. Figure S2: Estimated deposition curves in the airways of UV-Titan in exposed mice. (A) Estimated accumulated mass deposition curves in the airways of UV-Titan in exposed mice. (B) Estimated accumulated particle number deposition curves in the airways of UV-Titan in exposed mice. Figure S3. Basal startle reaction, in male (A) and female (B) offspring from dams exposed to ambient air or TiO2 during gestation. [file 1743-8977-7-16-S1.PDF]

Additional file 1, Particle and Fibre Toxicology:

Hougaard KS, Jackson P, Jensen KA, Sloth JJ, Löschner K, Larsen EH, Birkedal RK, Vibenholt A, Boisen AM, Wallin H, and Vogel U: ñEffects of prenatal exposure to surface-coated nanosized titanium dioxide (UV-Titan). A study in miceò.

Housing of animals:

Animals were housed in groups of 5 or 6 in white polypropylene cages (type III) with bedding (Jeluxyl HW 300/500, JELU-Werk J. Ehrler GmbH & Co.KG, Rosenberg, Germany). Soft paper wool (Enviro-Dri), paper tubes and small aspen wood blocks were added as enrichment (Lillico, Betchworth, UK), though removed during nursing. The environment in the animal facility was controlled (12 hour light-dark cycles with light starting at 6.00 a.m., temperature 21 ± 2°C, humidity 50 ± 5%, ventilation 13 air changes per hour) with access to food (Altromin 1324, Altromin GmbH, Lage, Germany) and tap water ad libitum.

Sample preparation for TEM analysis

Particles (ca. 0.05 mg/ml) were suspended in MilliQ-filtered de-ionized water by sonication using a Branson Sonifier S-450D (Branson Ultrasonics Corp., Danbury, CT, USA) equipped with a standard 13 mm disruptor horn. Sonication was performed on ice for 4 min with alternating 10 sec pulses and pauses at 10% amplitude. Samples of suspended particles were then immediately drawn by pipette and 1-2 droplets were placed onto the carbon side on holey carbon-coated Cu TEM-grids. The TEM-grids were placed on a glass petri-dish with filter-paper to absorb excess liquid leaving only a thin layer of particle suspension behind, which was allowed to dry in air for about 5 minutes before the TEM-grids were transferred for individual storage in a BEEM embedding capsules.

On-line particle exposure monitoring

Particle exposure was monitored using a GRIMM Sequential (Stepping) Mobility Particle Sizer (SMPS) consisting of a Long Electrostatic Classifier (Model No. 5.521) and a GRIMM Condensation Particle Counter (Model 5.400). The SMPS was operated in fast scan mode completing size distribution scans (9.8 to 874.8 nm) within 218 sec. Data were corrected for both Classifier and CPC efficiency using the software options. A 3.7 MBq Am-241 α-source (Model 5.521) was used for charge equilibration. Two impactors with nominal d_{50} cut-points of 1 185 and 1 082 µm were mounted at the DMA inlet resulting in an upper cut-point of 486 nm for SMPS data using the rutile density of 4.25 g/cm^3 (The Mineralogy Database 2010). Therefore, SMPS data for

particles larger than 486 nm were excluded. Data for particle sizes below 12.8 nm were also omitted due to insufficient clearance times between scans. Coarse particles (0.75 to >15 εm) were monitored with a GRIMM Dust Monitor (Model 1.105) at a resolution of 6 sec. Dust Monitor sizes were recalculated to log-transformed geometric mid-points assuming an upper channel cut-point at 32 εm. (Both instruments from Grimm Aerosol Technik GmbH & Co. KG (Ainring, Germany).

s

BAL preparation and analyses

BAL was performed four times with 0.8 ml 0.9% sterile saline and prepared as described (Saber *et al.* 2005). The total number of cells and of dead cells in BAL samples was determined in cell suspension B by NucleoCounter (NucleoCassetteTM ï 941-0002, Chemometec, Denmark) following standard procedures. Differential counts of macrophages, neutrophils, lymphocytes, eosinophils, and epithelial cells were determined by counting 200 cells in cell supernatant collected from 40 ìl cell suspension-A, centrifuged at 55´g for 4 min at Cytofuge 2 (StatSpin, Bie and Berntsen, Denmark). Slides were fixed with 96% ethanol and stained with May-Grünwald-Giemsa stain. All slides from both time points were randomized, blinded and scored on the same day. Total number of cells was calculated by combining data from differential cell counts with the total number of cells in BAL.

Table S1. Settings for the ICPMS measurements

Setting	Value
Forward power	1550 W
Plasma gas flow rate	15 L/min
Carrier gas flow rate	0.86 L/min
Make-up gas flow rate	0.32 L/min
He gas flow rate (CRC)	3.5 mL/min
Nebuliser	PFA concentric
Spraychamber	Water cooled double pass (15°C)
Lens voltage	3.2 V
Mass resolution	0.8 amu
Integration time	200 ms /isotope
Analyte isotopes	^{49}Ti, ^{50}Ti (SCP Science, Courtaboeuf, France)
Internal standard	^{45}Sc (CPI International, Amsterdam, The Netherlands)
Calibration	External with internal standardization

Table S2: Pregnancy and litter data

	Control	Exposed
No. time mated females	22	23
No. females delivering litters	13	14
Maternal weight gain, GD4-18 (g)	10.5±3.07	10.1±3.21
Maternal weight gain, PND1-23 (g)	3.56±0.28	3.17±0.44
Gestation length (days)	20.2±0.10	20.0±0.00
Implantations, NP females	0	0
Implantaions, pregnant females	8.33±0.63	8.64±0.31
Implantation loss (%)	20.02±4.72	21.96±3.34
Live pups per litter, PND1	6.15±0.39	6.5±0.40
Observed dead pups, PND1	0.08±0.08	0.29±0.16
Pups dead during lactation (*)	0.31±0.24	0.71±0.30
% females in litters	45±4	45±3
Birth weight, females (g)	1.30±0.04	1.31±0.03
Birth weight, males (g)	1.31±0.04	1.33±0.03
Weight gain, females, PND1-23 (g)	7.12±0.26	7.00±0.32
Weight gain, males, PND1-23 (g)	7.41±0.27	7.38±0.26

Females that did not give birth were sacrificed on PND3, and littering females at PND25. Data are expressed as mean±SEM. NP: Nonpregnant time-mated females. (*) $0.05 < p < 0.01$.

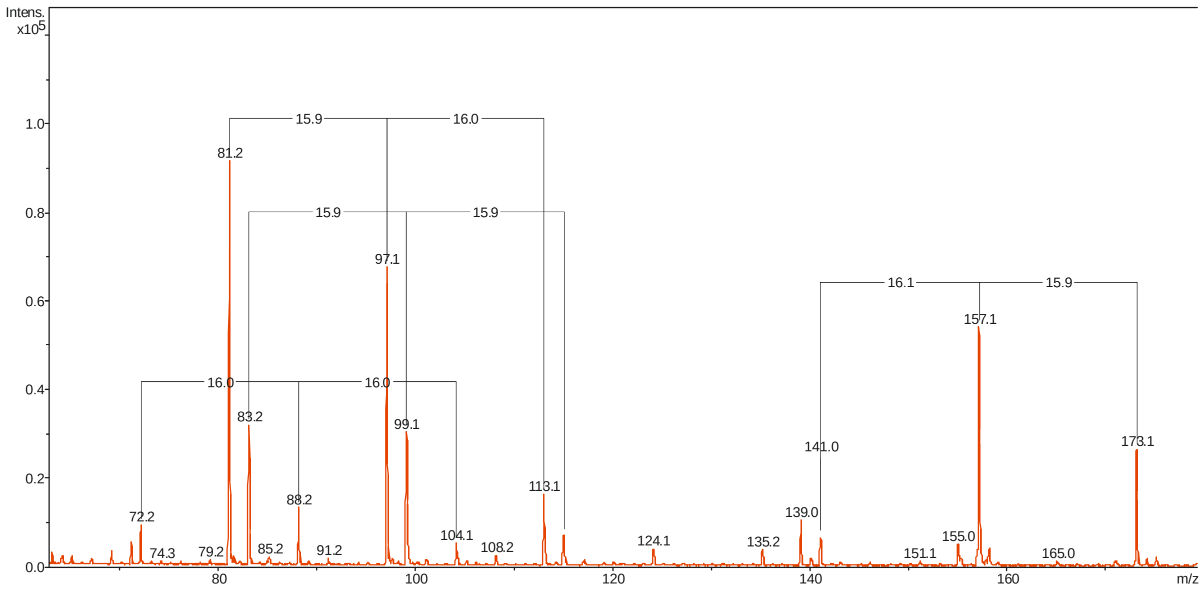

Figure S1. MALDI-TOF spectrum of methanol extract of UV-titan 181

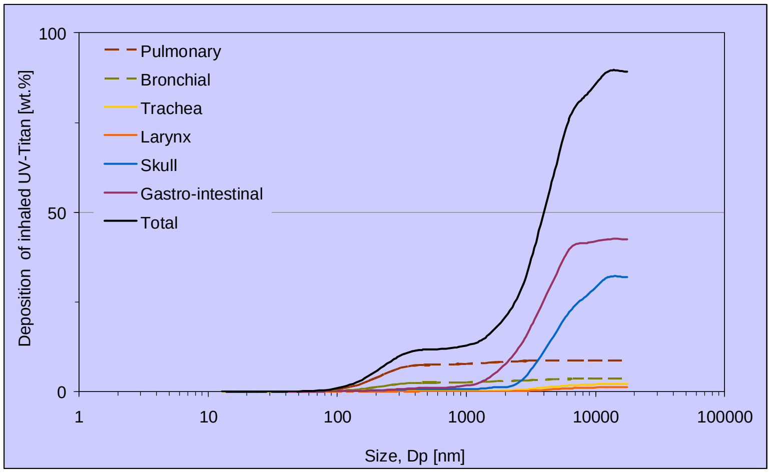

Figure S2A

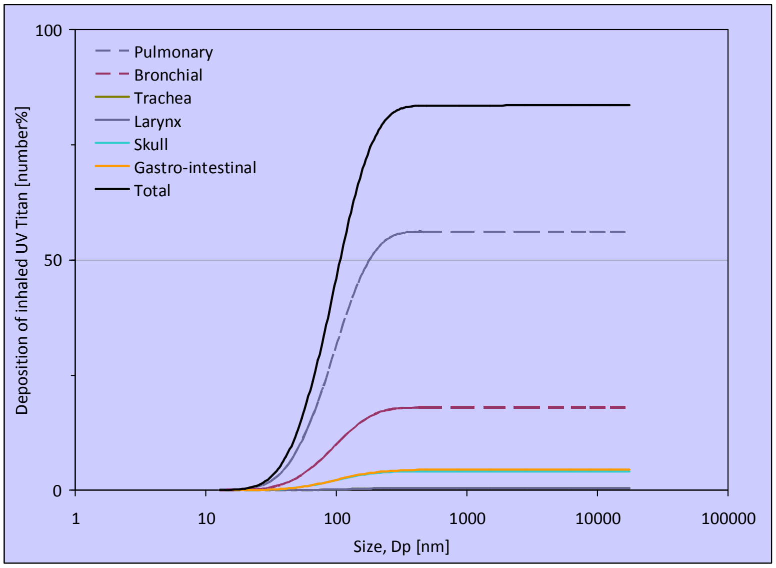

Figure S2B

Estimated deposition curves in the airways of UV-Titan in exposed mice. Deposition curves are based on the deposition model applied in (Jacobsen *et al.* 2009)). A: Estimated accumulated mass deposition curves in the airways of UV-Titan in exposed mice. B: Estimated accumulated particle number deposition curves in the airways of UV-Titan in exposed mice.

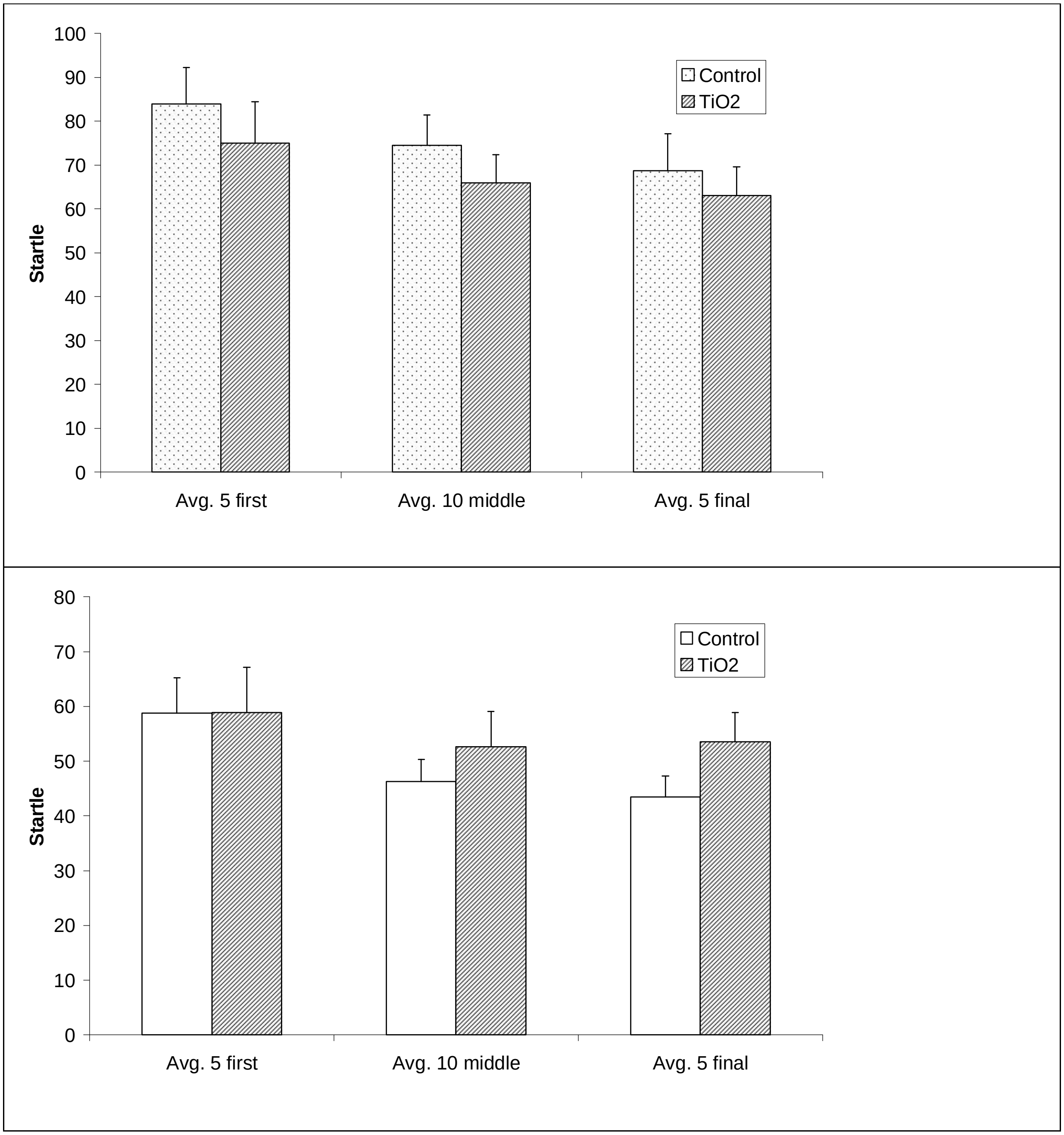

Figure S3A and S3B

Basal startle reaction in male (A) and female (B) offspring from dams exposed to ambient air or TiO_2 during gestation. Mean±SEM, n = 10-14. The three columns depict the average of basal startle following the average reaction to the initial 5 noise bursts of 120 dB(A) (Avg. 5 first), the middle 10 noise bursts (Avg. 10 middle) and the final 5 noise bursts (Avg. 5 final).

Reference List

Jacobsen NR, Moller P, Jensen KA, Vogel U, Ladefoged O, Loft S & Wallin H 12-1-2009 Lung inflammation and genotoxicity following pulmonary exposure to nanoparticles in ApoE-/- mice. *Part Fibre.Toxicol.* **6:2.** 2.

Saber AT, Bornholdt J, Dybdahl M, Sharma AK, Loft S, Vogel U & Wallin H 2005 Tumor necrosis factor is not required for particle-induced genotoxicity and pulmonary inflammation. *Arch Toxicol.* **79** 177-182.

The Mineralogy Database. Rutile mineral data. 15-2-2010. Internet Communication.
